# Expressions of loss predict aggressive comments on Twitter among gang-involved youth in Chicago

**DOI:** 10.1038/s41746-018-0020-x

**Published:** 2018-03-28

**Authors:** Desmond Upton Patton, Owen Rambow, Jonathan Auerbach, Kevin Li, William Frey

**Affiliations:** Columbia School of Social Work, Columbia University, 1255 Amsterdam Avenue, New York, NY 10027-5927 USA

**Keywords:** Sociology, Social sciences, Society

## Abstract

Recent studies suggest social media shapes the transmission of firearm violence in high-poverty, urban neighborhoods. However, the exact pathways by which content on social media becomes threatening has not been studied. We consider a dataset of tweets by gang-involved Chicago youth that are coded for expressions of aggression and/or loss. Using a permutation test and mixed-effects log linear regression, we find that aggression and loss tweets do not occur randomly, and furthermore that in a 2-day window after loss expressions we find an increase in aggressive tweets. We discuss implications for intervention.

## Introduction

Increasing evidence suggests that social media plays a role in the transmission of firearm violence in high-poverty, urban neighborhoods.^1,2^ However, the exact pathways by which content on social media becomes threatening remains unclear. In a qualitative study of Twitter communication among gang-involved youth in Chicago, we identified two main themes in the labeled dataset, *Loss* and *Aggression*, and observed loss expression generally preceding aggressive posts.^3^ We support our earlier qualitative observations by testing quantitatively whether aggression and loss are randomly distributed in the dataset, and whether there is a temporal relationship between the two categories of tweets.

## Results

Loss and aggression are not randomly tweeted by users, and low *p*-values in the permutation test are evidence of a sequential relationship between tweet labels. We find strong evidence (Table 1) that loss and aggression tweets cluster since the *p*-values are lower than .05.

**Table 1:** Permutation test results

Lagging label	Leading label		
	Aggression	Loss	Other
Aggression	0	0.217	1
Loss	0.995	0	0.345
Other	0.984	1	0.03

**Fig. 1:**
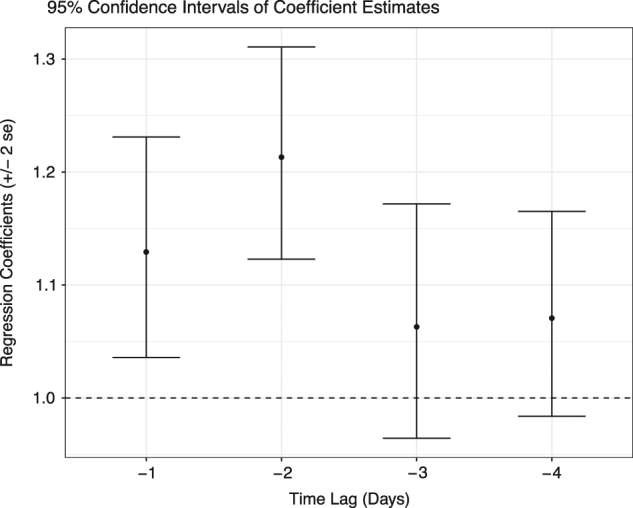
Confidence intervals of coefficient estimates

Loss tweets are significantly predictive of aggressive tweets. The model states that loss tweets are followed by a 13% increase in aggressive tweets the next day. A 95% confidence interval is 3.5 to 23%. Two days after a loss tweet, we see a 21% increase in the expected number of aggressive tweets. A 95% confidence interval is 12–30% (see Fig. 1). Loss tweets do not appear predictive of aggressive tweets 3 or more days out.

## Discussion

This brief highlights the value of leveraging social media to understand loss and aggression in Twitter posts from youth and those who identify as gang-involved in Chicago and offers two meaningful findings: (1) loss and aggression tweets are not random and (2) posting a loss tweet increases the likelihood of an aggressive tweet in the next 2 days. These findings confirm prior qualitative observations and theory that loss and aggressive behavior on Twitter are connected.^1,4,5^ The findings underscore the idea that online behavior is rooted in offline events.^5^ The multiple day opening provides an opportunity to provide resources to young people before their grieving turns to aggression. The time between loss and aggressive tweets could be used to prevent offline violence by providing grief counseling and other mental health services as well as traditional clinical practices.

## Methods

We used a corpus of publicly available Twitter communications from a self-identified gang member and users in her Twitter network.^3^ We increased the size of the original data by including the last 200 tweets in a chosen time window from the Twitter network of the 14 users who were most communicative with the original users. Our dataset consists of 2256 tweets from 235 unique users. The tweets were coded as “loss”, “aggression”, or “other.” Loss was defined as expressions of grief, trauma, or sadness or mentions of death or incarceration. Aggression was defined as insults, threats, mentions of physical violence, or of wanting retribution. Posts identified as “other” did not contain features involved in aggression or loss. The number of tweets per account ranged from 1 to 200, with an average of 10. One hundred and eighty five users tweeted at least one aggressive or loss tweet. Twenty five of the users made a transition from loss to aggression at least once.

First, we evaluated how users transition between loss and aggression in sequential tweets. We considered the null hypothesis that aggressive and loss content is tweeted by users at random, and *p*-values reflecting the weight of the evidence supporting this hypothesis were approximated using a permutation test. Specifically, we constructed a 3 × 3 transition matrix for each user that contained the observed frequency of the two labels of two consecutive tweets from the same user within a 24-h window. The test statistic is the mean matrix, obtained by averaging the transition frequencies across users.

We randomly permuted the order of the tweets and reconstructed the mean matrix 1000 times.^6^ The number of permutations that yield larger mean transitions than those observed divided by 1000 yielded a 3 × 3 matrix of *p*-values (see Table 1).

Second, we established that the number of loss tweets is predictive of the number of aggressive tweets within a subsequent time window. We fit a mixed-effects log linear model to predict the daily number of aggressive tweets.^7^ The fixed effects or predictors are the daily number of loss tweets in the previous 1, 2, 3, and 4 days. A random user intercept accounts for the baseline number of aggressive tweets per user. The regression coefficients were estimated using maximum likelihood. The estimates and their standard errors are presented in Fig. 1. There are four regression coefficients, representing the increase in the number of aggressive tweets expected for each of the four time intervals.

### Data availability

Our social media data will be placed in a publicly available repository housed at Columbia University.
